# Flow cytometric quantification of neutral lipids in a human skin stem cell-derived model of NASH

**DOI:** 10.1016/j.mex.2020.101068

**Published:** 2020-09-19

**Authors:** Joost Boeckmans, Alessandra Natale, Matthias Rombaut, Karolien Buyl, Tamara Vanhaecke, Vera Rogiers, Robim M Rodrigues, Joery De Kock

**Affiliations:** Department of In Vitro Toxicology and Dermato-Cosmetology, Faculty of Medicine and Pharmacy, Vrije Universiteit Brussel, Laarbeeklaan 103, Brussels 1090, Belgium

**Keywords:** Flow cytometry, Steatosis, Non-alcoholic steatohepatitis (NASH), Adult stem cells, Human skin-derived precursors (HSKP), Preclinical drug testing, *In vitro*

## Abstract

Non-alcoholic steatohepatitis (NASH) is a severe chronic liver disease that affects 3 to 5 percent of the world population. It is characterized by hepatic lipid accumulation and inflammation and can progress towards fibrosis, cirrhosis and hepatocellular carcinoma. Until today, no drug has been approved for the treatment of NASH. This delay relates to the complex pathogenesis of NASH and also to a lack of appropriate predictive preclinical testing systems. Furthermore, the human specificity of the NASH pathology hampers *a fortiori* clinical translation of animal studies.

Therefore, we recently employed human skin-derived precursors (hSKP) differentiated to hepatocyte-like cells (hSKP-HPC) as a human-relevant cell source for modelling NASH *in vitro*. Using this *in vitro* NASH model, it was possible to test novel drugs being developed for anti-NASH therapy, such as elafibranor. Since steatosis is an important aspect of NASH and multiple drugs are being developed to decelerate and reduce lipid accumulation in the liver, we optimized a flow cytometric method for quantifying neutral lipids in ‘NASH’-triggered hSKP-HPC. This methodology enables efficient identification of anti-steatotic properties of new medicines.

• NASH-triggered hSKP-HPC robustly accumulate lipids intracellularly.

• Flow cytometric quantification of neutral lipids in NASH-triggered hSKP-HPC allows for accurate determination of the steatotic response.

• This method enables efficient identification of potential anti-steatotic drugs in a human-specific model

## Graphical abstract

**Figure:**
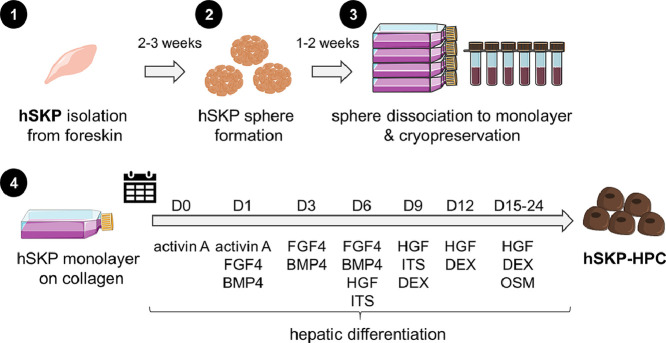

Specifications Table

**Table:** 

Subject Area:	Pharmacology, Toxicology and Pharmaceutical Science
More specific subject area:	Preclinical drug testing
Method name:	Flow cytometric quantification of neutral lipids in human skin-derived hepatic cells
Name and reference of original method:	R. M. Rodrigues, S. Branson, V. De Boe et al., *In vitro* assessment of drug-induced liver steatosis based on human dermal stem cell-derived hepatic cells, Arch. Toxicol., 90 (3) (2016) 677–689. M. T. Donato, A. Martínez-Romero, N. Jiménez et al., Cytometric analysis for drug-induced steatosis in HepG2 cells, Chem. Biol. Interact. 181 (2009) 417–423.
Resource availability:	*N/A.*

## Method details

Non-alcoholic fatty liver disease (NAFLD) is a highly prevalent chronic liver disease that is closely associated with the metabolic syndrome [1]. NAFLD encompasses a spectrum of diseases ranging from liver steatosis to severe non-alcoholic steatohepatitis (NASH), fibrosis, cirrhosis and hepatocellular carcinoma. NASH is characterized by hepatic lipid accumulation and inflammation and fulfills a key role in NAFLD since it is considered as the tipping point to the latter life-threatening conditions [2]. At present, no drug has been approved to treat or cure NASH [3]. Liver steatosis is an important parameter of NASH  [4,5], hence, the majority of newly-developed anti-NASH drugs focuses, apart from tackling hepatic inflammation, also on liver steatosis. Animal models are traditionally used for preclinical NASH research. Yet, important interspecies differences exist between rodents and humans with regard to the development of NASH and investigation of anti-NASH therapies. For example, when peroxisome proliferator-activated receptor alpha (PPAR-α) agonists, which are drugs that are used for treating dyslipidemia, are administered to rodents, these animals develop liver cancer [6]. Considering that this complex disease could be solved using PPAR agonists highlights the need for human-relevant models in early anti-NASH drug development [3].

Our group has developed a protocol for differentiating human skin-derived precursors (hSKPs) to hepatic cells (hSKP-HPC) by mimicking the liver embryogenesis *in vitro*. hSKP-HPC exhibit a mixed phenotype of mature (*ALB*) and immature (*EPCAM, NCAM2, PROM1*) hepatocytes [7]. The applicability of hSKP-HPC has been earlier demonstrated for drug-induced liver injury (DILI) by using the reference compounds acetaminophen [7], sodium valproate [8] and amiodarone [9], respectively inducing acute liver injury, steatosis and phospholipidosis. hSKP-HPC have been also employed for *in vitro* modelling of metabolically-induced liver steatosis and NASH [10]. When hSKP-HPC were exposed to fatty acids, insulin, glucose and inflammatory cytokines, these cells massively accumulated lipids and secreted inflammatory cytokines characteristic to NASH. When these ‘NASH’-triggered cells were challenged with an anti-NASH drug under development (*i.e.* elafibranor, a PPAR-α/δ agonist), clear anti-inflammatory and anti-steatotic responses could be observed, of which the latter was mediated by different cellular processes including fatty acid transport, endoplasmic reticulum stress and *de novo* lipogenesis. These successful applications are a consequence of the fact that hSKPs acquire a strong lipid metabolism upon hepatic differentiation to hSKP-HPC. Furthermore, hSKP-HPC recently showed to mimic the anti-steatotic responses induced by a series of PPAR agonists in a similar way as primary human hepatocytes, being the gold standard for hepatic *in vitro* modelling, emphasizing the position of hSKP-HPC for modelling NASH *in vitro* [11]. Considering the restricted availability and fragility of primary human hepatocytes, we optimized a flow cytometric method to quantify the anti-steatotic response induced by potential anti-NASH pharmaceuticals in hSKP-HPC. This methodology can be implemented in early preclinical drug development for testing of anti-steatotic properties of new chemical entities (NCE) in a human-relevant way.

## hSKP-HPC culture

**Fig. 1:**
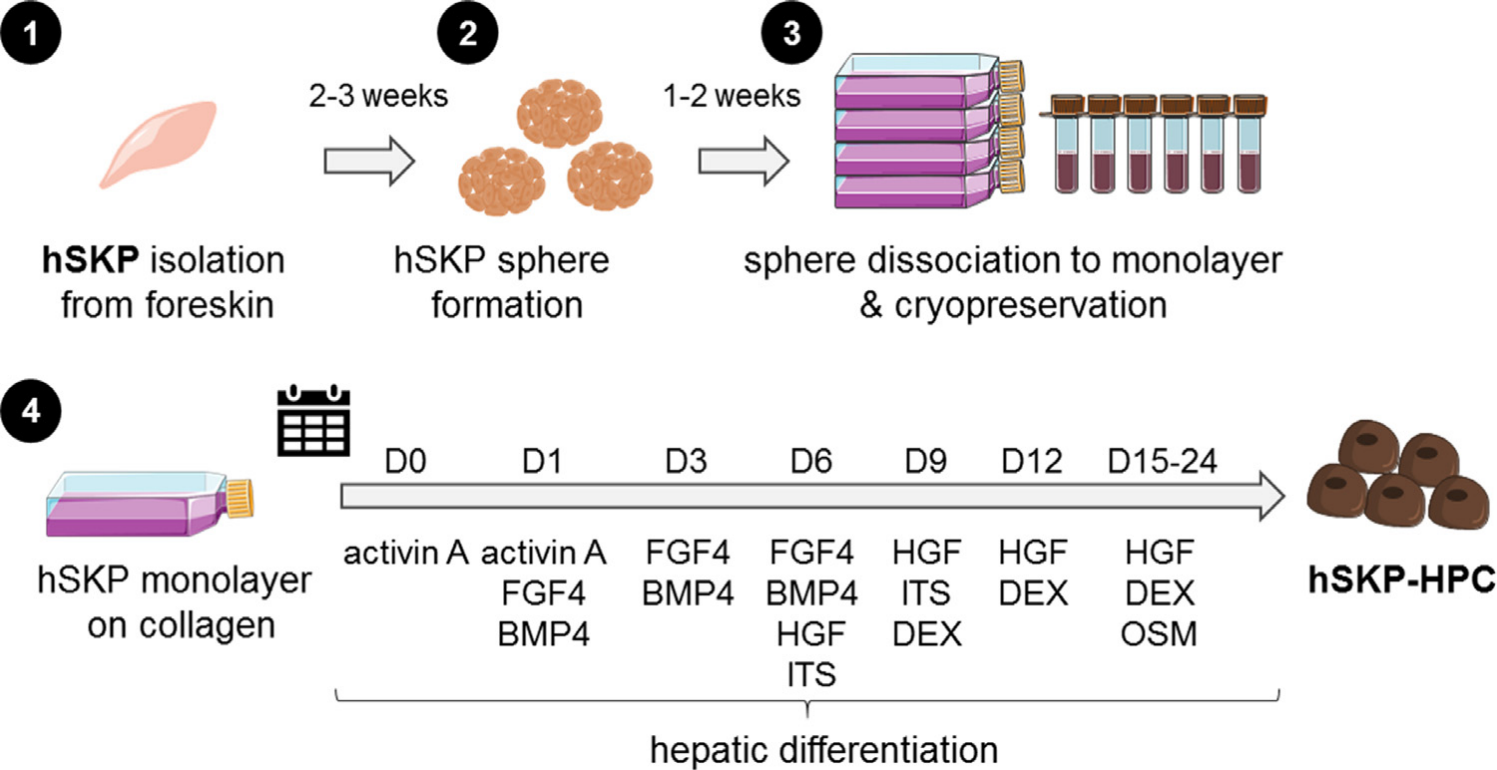
hSKP isolation and differentiation to hSKP-HPC. (1) hSKP are isolated from human foreskin. (2) hSKP form spheres in a medium containing EGF and FGF2. (3) Spheres are digested to a 2 dimensional (D) culture, subcultured and cryopreserved. (4) Hepatic differentiation to hSKP-HPC. [Figure abbreviations: BMP: bone morphogenic factor; DEX: dexamethasone; FGF: fibroblast growth factor; HGF: hepatocyte growth factor; hSKP: human skin-derived precursor, hSKP-HPC: human skin-derived precursor hepatocyte progenitor cell, ITS: insulin transferrin selenium; OSM: oncostatin M].

hSKPs are isolated and differentiated as earlier reported [7,12]. Briefly, hSKPs are isolated from human (fore)skin dermis using a series of enzymatic and mechanical steps. hSKPs aggregate and form spheres through clonal expansion in a medium containing epidermal growth factor (EGF) and fibroblast growth factor 2 (FGF2). After 2–3 weeks, the spheres are dissociated into single cells and grown in adherent monolayer cultures. Cells cultured in monolayers can be further expanded and cryopreserved. hSKPs are differentiated on rat tail collagen type I-coated cell culture recipients and are required to reach 90–95% confluence before hepatic differentiation is initiated. After 24 days of hepatic differentiation, hSKP-HPCs are obtained. A graphical representation of the hSKP culture and hepatic differentiation to hSKP-HPC is given in Fig. 1. For the purpose of this flow cytometric analysis, the cells can be cultured in either 6- or 24-multiwell (MW) plates. One well from a 6-MW plate or four wells from a 24-MW plate are sufficient per sample for performing the analysis.

## Establishment of ‘hSKP-HPC NASH’ and drug exposure

### Preparation of stock solutions

- Bovine serum albumin (BSA) (7% (m/v)): Transfer 420,0 mg fatty acid free BSA (Sigma-Aldrich) in a 50 mL conical tube (Falcon BD) with 6.0 mL Day 24 (D_24_) medium. Vortex the tube during 30 s. and put on a 37 °C warm water bath for 15 min. A 50 mL conical tube is required due to the bulky nature of fatty acid free BSA. This solution is freshly prepared on the day of exposure.
- Sodium oleate (3.25 mM): Calculate the required amount of sodium oleate (Sigma-Aldrich) to obtain a 50-fold concentrated solution (*e.g.* 3.958 mg for 4.0 mL stock solution) according to the final 65 µM concentration in culture. Quantitatively transfer the sodium oleate in an appropriate volume fatty acid free BSA 7% (m/v) and vortex during 2 min. Ensure that the BSA solution is pre-warmed to 37 °C and put the tube on a warm water bath for another 15 min. Evaluate whether the solution is clear and if not, repeat the vortex step and put the tube back on the warm water bath. This stock solution is freshly prepared on the day of exposure.
- Palmitic acid (90 mM): 11.539 mg palmitic acid (Sigma-Aldrich) is dissolved in 500 µL dimethylsulfoxide (DMSO) (Sigma-Aldrich) (or equivalent) in a 15 mL conical tube (Falcon BD) to obtain a 2000-fold palmitic acid stock solution according to the final concentration of 45 µM in culture. Vortex the tube during 2 min. to ensure that palmitic acid is completely dissolved. This stock solution can be stored for 6 months at - 20 °C.
- IL-1β (25 µg/mL): IL-1β (Peprotech) is dissolved in phosphate buffered saline (PBS) as a 1:1000 solution according to the final concentration of 25 ng/mL in culture. This stock solution can be stored for 6 months at - 20 °C.
- TGF-β (5 µg/mL): TGF-β (Peprotech) is dissolved in PBS (5 µg/mL) as a 1:625 solution according to the final concentration of 8 ng/mL in culture. This stock solution can be stored for 6 months at - 20 °C.
- TNF-α (20 µg/mL): TNF-α (Prospec) is dissolved in PBS as a 1:400 solution according to the final concentration of 50 ng/mL in culture. This stock solution can be stored for 6 months at - 20 °C.
- Insulin (50 µM): 1.147 mg bovine pancreas insulin (Sigma-Aldrich) is dissolved in 4.0 mL basal medium (or equivalent) [7] in a 15 mL conical tube to obtain a 500-fold insulin stock solution according to the final concentration of 100 nM in culture. Vortex the tube during 2 min. and put for 10 min. on a 37 °C warm water bath.
- All testing compounds can be dissolved to 1:1000 solutions in DMSO according to the final concentration in culture medium to avoid the need of multiple vehicle control samples. Elafibranor (AdooQ Bioscience) at a concentration of 30 µM can serve as a positive control [10,13].

### Preparation of exposure solutions

hSKP-HPC control media are composed of the medium used for the final steps of the differentiation (D_24_ medium) [7] supplemented with fatty acid free BSA (0.14% (m/v)) and DMSO (0.15% (v/v)).

hSKP-HPC ‘NASH’ media are composed of D_24_ medium supplemented with glucose (Sigma-Aldrich) ((4.5 mg/mL) together with insulin (100 nM), sodium oleate (65 µM), IL-1β (25 ng/mL), TGF-β (8 ng/mL) and TNF-α (50 ng/mL). Palmitic acid needs to be 37 °C pre-warmed and added quickly to 37 °C pre-warmed medium, directly followed by a 30 s. vortex step to avoid precipitation. Hereafter either the compound or its vehicle (DMSO) can be added (1:1000). Note: in this protocol the compound is dissolved to obtain a 1000-fold concentrated stock solution. Some compounds are less soluble, which implies that also higher DMSO concentrations need to be added to the control and vehicle media, however final DMSO concentration cannot exceed 0,5% (v/v).

All solutions are filtered through a medium filter with 200 µM pore size before exposure to the cells.

### Exposure of hSKP-HPC

Expose the cells to 500 µL or 1500 µL media per well of the 24- or 6- MW plates, respectively. Incubate hSKP-HPC for 24 h at 37 °C in a humidified incubator with 5% CO_2_.

## Flow cytometric analysis of neutral lipids

### Harvesting of hSKP-HPC and dissociation into single cells

After 24 h of incubation, the media are gently removed from the cells and replaced by either 200 µL (24-MW plate format) or 500 µL (6-MW plate format) trypsin solution (TrypLE reagent, Thermo Fisher Scientific) per well. Then, the cells are incubated for 10 min. at 37 °C in a humidified incubator with 5% CO_2_. After 10 min., cell detachment is evaluated under a phase contrast microscope (Nikon Eclipse) at 10 x magnification. If the cells do not come loose after 10 min., the culture plate is gently tapped on the sides and incubated for 5 more min. Subsequently, 500 µL (24-MW format) or 1500 µL (6-MW format) 37 °C pre-warmed PBS is added to each well to stop the action of the TrypLE reagent. hSKP-HPC are resuspended into single cells by gentle pipetting and are collected in a 15 mL conical tube. The wells are rinsed with 37 °C pre-warmed PBS using the same volumes. One additional control sample is included for setting up the flow cytometer.

### Sample preparation

The conical 15 mL tubes containing hSKP-HPC are centrifuged during 5 min at 470 g. After 5 min., the supernatant is gently aspirated using a 5 mL pipet and the pellet is resuspended with 1 mL PBS containing 2 µM BODIPY™ 493/503 neutral lipid dye (Thermo Fisher Scientific). To measure events that correspond to actual cells, cell nuclei are labelled with Hoechst 33,342 (Life Technologies) by adding 1 µL of the dye to the cell suspension (1:1000) and gently pipetting up and down, 5 min. before measuring with the flow cytometer. One sample is resuspended in 1 mL PBS without any dyes for setting up the flow cytometer. From this point, the cells are kept on ice and in the dark. Right before measuring, 400 µL of the sample is diluted with 3600 µL cold PBS. The remaining 400 µL allows for obtaining a technical replicate.

### Instrument settings

**Fig. 2:**
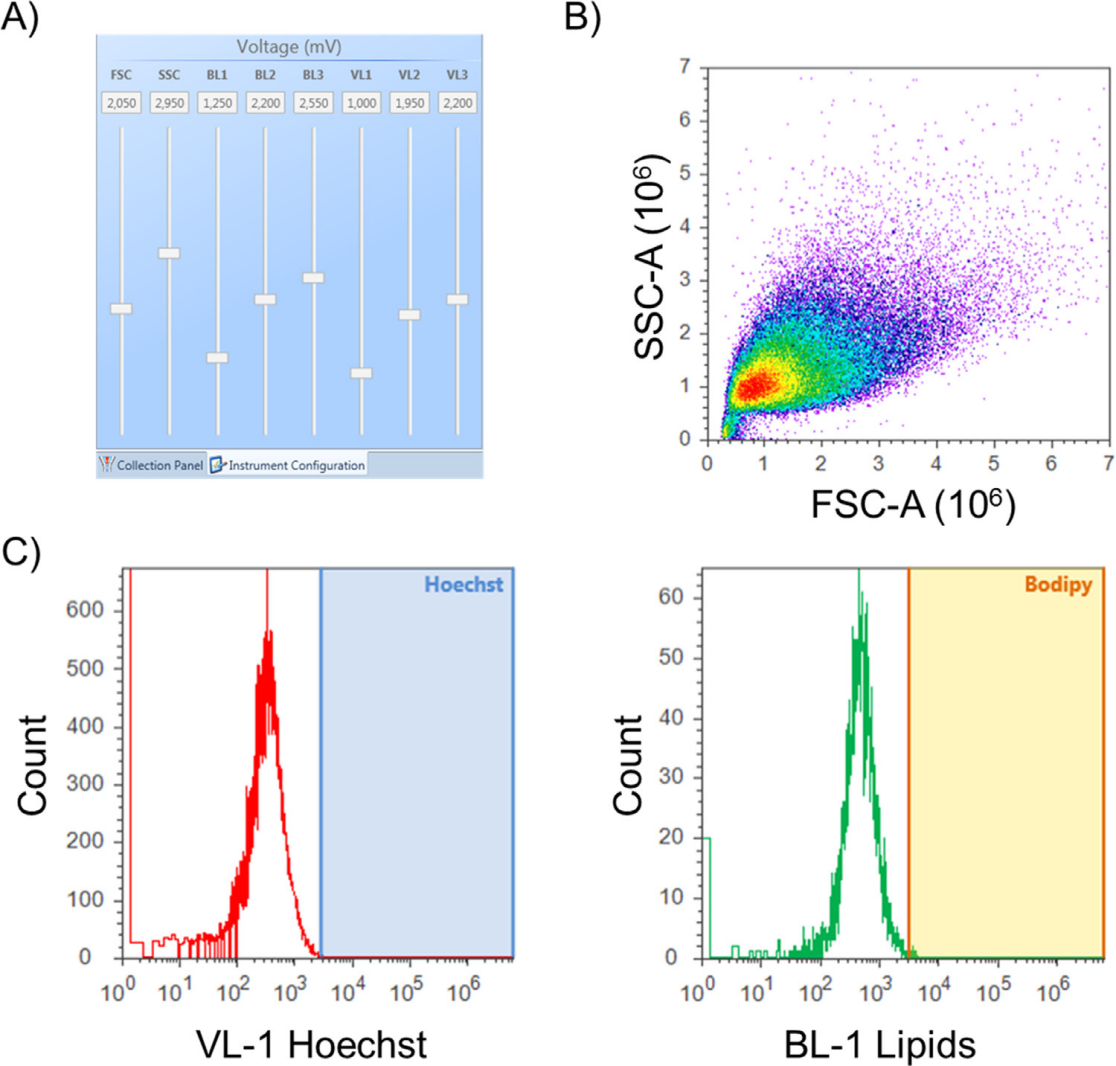
Setting up the flow cytometer A) Voltages of forward-and side- scatter need to be adjusted for B) obtaining central spreading of the cell population in the dot plot. C) VL-1 and BL-1 channel signals for the unstained sample are ideally adjusted between 10^3^ and 10^4^ relative fluorescence to ensure a wide enough measuring range. [Figure abbreviations: BL: blue laser, FSC-A: forward scatter area, SSC-A: side scatter area, VL: violet laser].

The Attune^Ⓡ^ Acoustic Focusing Cytometer (Life Technologies) is used for flow cytometric analysis. The flow cytometer is set at a flow rate of 500 µL per second with a maximum detection of 100.000 events. The specific fluorescence detection channels are chosen for BODIPY™ 493/503 (blue laser (BL)−1 channel) and Hoechst 33,342 (violet laser (VL)−1 channel). After quality control of the flow cytometer according to the manufacturer's instructions, the unstained control sample is ran for adjusting the voltages (Fig. 2A) of the forward scatter (FSC), side scatter (SSC), BL-1 and VL-1 channels. The voltages of FSC and SSC need to be adjusted so that the cell population spreads in the main area of the dot plot, and can be distinguished from the debris material (Fig. 2B). Fig. 2C shows the unspecific signals generated on the VL-1 and BL-1 channels from an unstained sample. Voltages are ideally set to obtain unspecific relative fluorescence signals from maximal 10³ à 10^4^ to allow for a wide enough measuring range (especially for the BL-1 channel to measure a shift in neutral lipid load). Hereafter, gates (‘Hoechst’, blue and ‘Bodipy’, yellow) are set in order to only measure signals derived from the specific dyes.

### Data analysis

**Fig. 3:**
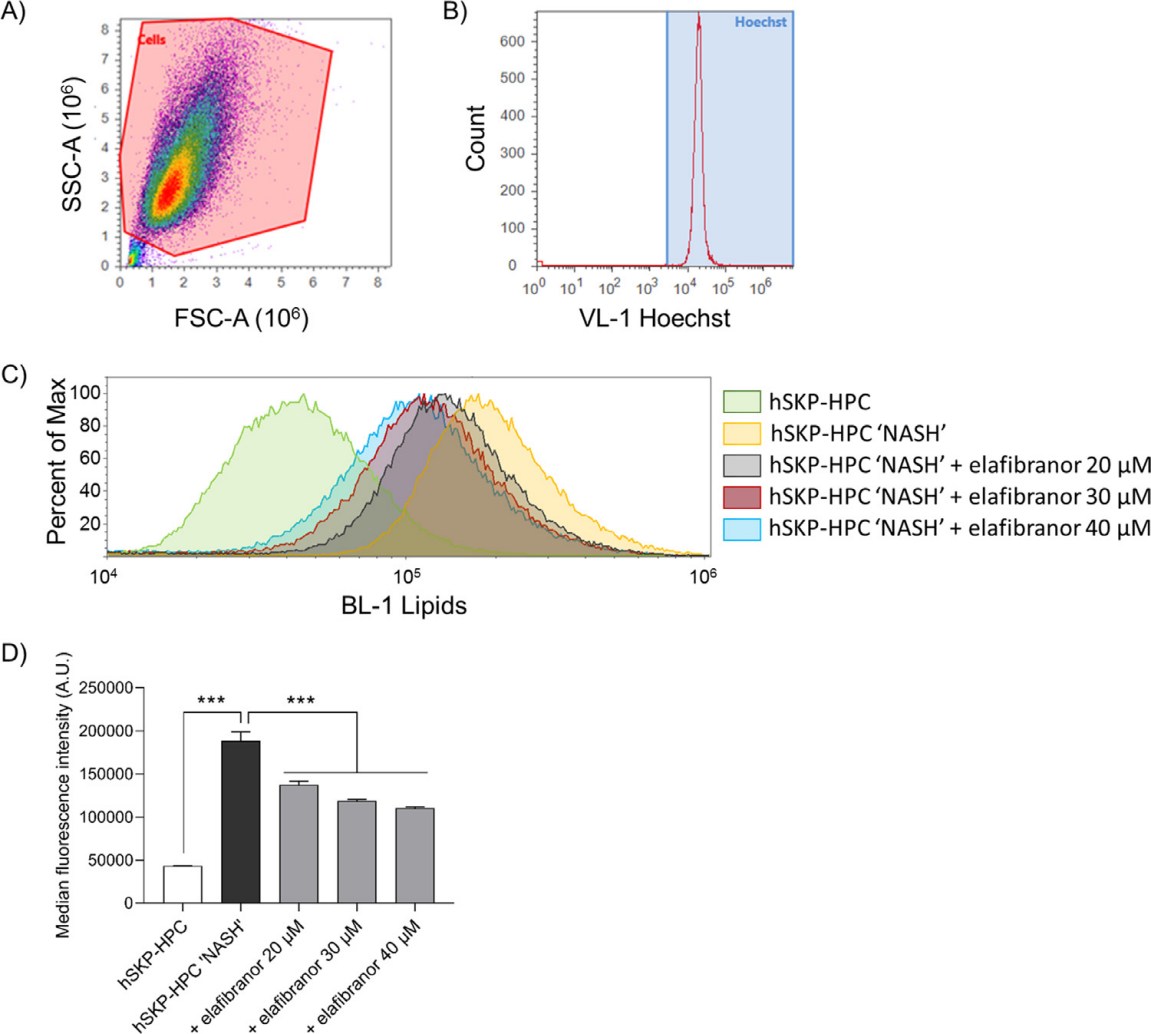
Data generation. A) The cell population is selected based on the FSC and SSC, while the debris is excluded. B) The gate of BODIPY-positive cells needs to be set as a function of Hoechst-33,342 positive cells. C) The histogram shows a shift in the neutral lipids from the hSKP-HPC control (green) to hSKP-HPC ‘NASH’ (yellow) sample. This shift gradually reverts back to the control sample upon exposure to increasing concentrations of elafibranor. D) Bar graph of the median fluorescence intensities, obtained from the flow cytometric analysis, shows that hSKP-HPC exposed to ‘NASH’ triggers reveal an important intracellular increase in neutral lipids. Elafibranor dose-dependently reduces the increased lipid load. [***: One-way ANOVA with *post hoc* Sidak's multiple comparisons test, *p* ≤ 0.001 (*n* = 3)] [Figure abbreviations: A.U.: arbitrary units, BL: blue laser, FSC-A: forward scatter area, hSKP-HPC: human skin-derived precursor hepatocyte progenitor cell, NASH: non-alcoholic steatohepatitis, SSC-A: side scatter area, VL: violet laser].

Debris, which can be observed as an accumulation of events having almost baseline forward- and side scatters, is excluded from the analysis (Fig. 3A). Hereafter, the BODIPY™ 493/503 neutral lipid dye signal is set as a function of Hoechst 33,342-positive cells (Fig. 3B). An induction in intensity of BODIPY™ 493/503 is observed in hSKP-HPC exposed to ‘NASH’ triggers compared to control hSKP-HPC (Fig. 3C). Exposure to elafibranor induces a shift to the left, indicating a decrease in intracellular lipids.

Quantification of this observation can be done by plotting the median fluorescence signals for each condition. As an example, Fig. 3D shows a significant induction in BODIPY™ 493/503 intensity in hSKP-HPC triggered with ‘NASH’ compounds, which decreases upon increasing elafibranor concentrations. As such, this method enables the testing of anti-steatotic properties of potential anti-NASH compounds using a human-relevant *in vitro* model.

## Declaration of Competing Interest

The authors declare that they have no known competing financial interests or personal relationships that could have appeared to influence the work reported in this paper.
